# Relationship between Children’s Independent Activities and the Built Environment of Outdoor Activity Space in Residential Neighborhoods: A Case Study of Nanjing

**DOI:** 10.3390/ijerph19169860

**Published:** 2022-08-10

**Authors:** Yang Zhou, Meng Wang, Siming Lin, Caiyun Qian

**Affiliations:** School of Architecture, Nanjing Tech University, Nanjing 211800, China

**Keywords:** residential neighborhood, children, children’s independent activity, outdoor space

## Abstract

Children are a vulnerable population that is frequently overlooked in urban planning. The spatial demands of children are garnering broader consideration in the development of public spaces in cities as efforts to promote child-friendly cities. Children’s independent activities (CIAs) during childhood are undeniably beneficial to their physical and mental health. Residential areas are the main places for children’s daily activities. Building a suitable outdoor activity space in the community for children’s recreation is an essential foundation for improving CIAs and promoting the development of child-friendly neighborhoods. A sample of 15 typical children’s outdoor activity spaces in residential neighborhoods of Nanjing, China, was selected for the study to observe and record CIAs. The built environment indicators of residential outdoor spaces were extracted, and correlation analysis was employed to investigate the residential outdoor space elements relevant to CIAs. The results indicated that at the site level, higher percentages of tree coverage and soft paving enhanced CIAs, while high functional mix inhibited them. Additionally, gated communities, top-notch sanitation, secure facilities, neighborhoods with higher residential densities, and a diversity of activity facilities all stimulated children to engage in independent activities. Furthermore, questionnaires for the guardian indicated that they placed a high priority on site safety, and that waterfront areas and activity sites where incidents had occurred decreased parents’ willingness to allow participation in CIAs, whereas safety education or the use of positioning devices may promote CIAs. Based on the above results, we proposed appropriate adaptations for places in residential neighborhoods. The study expects to create a higher quality environment in residential neighborhoods for children to play in public spaces and provide beneficial help to improve the child-friendly neighborhood.

## 1. Introduction

Children’s independent activities (CIAs), defined as the freedom to play around their own neighborhood independently or with their peers without adult supervision, are a genuine representation of children’s mobility [1]. It is also a fundamental approach for children to learn about the urban environment as they grow [2]. The signing of Convention on the Rights of the Child in 1990 brought widespread attention to children’s issues. In 1996, UNICEF and UN-Habitat formally launched the Child Friendly Cities Initiatives at the second United Nations Conference on Human Settlements (Habitat II) to create a system of urban governance that would hear the voices of children and fulfill their needs, priorities and rights [3]. Since then, a series of international declarations have been signed and a special committee has been established for children’s rights. Although there have been ongoing improvements to the child welfare and children’s rights systems, fewer substantive changes have occurred in the status of children’s rights.

With the development of urbanization, urban motorized traffic steadily encroaches on traditional street areas, and urban development initiatives shrink children’s outdoor space. The proportion and the spectrum of CIAs have become an irreversible fact. In China, the situation is perhaps even less optimistic. According to data from China’s seventh national census, there are approximately 250 million children under the age of 14, accounting for 17.95% of the country’s total population, and the number of children has also increased over the last decade. However, there is a dearth of variety and a small number of public outdoor areas in cities that are appropriate for children’s activities. The amount of time children spend outside is drastically reduced, and their rights and interests of space are gradually taken away. According to the Assessment Report on China’s Children and Youth Physical Fitness Index (2017), the evaluation score for children’s physical activity level is just 11.4 out of 100. The trend of motorization of children’s travel, indoor and static activities has triggered problems such as childhood obesity, impaired concentration, and mood swings, all of which have seriously affected children’s physical and mental health. Complicated reasons have conspired to bring forth these circumstances. Parents’ concern about the potential risks of children’s activity space, the unfriendly travel environment for children, the alteration of recreational activities due to the rapid development of electronic media, and the increasing pressure of schoolwork, all exert a negative influence on CIAs as well as their physical and mental health.

The role of CIAs in promoting healthy development has been demonstrated in many studies. Comparative studies have revealed that independent children tend to be more active and more physically active than those accompanied by parents [4,5]. In addition to encouraging children to become more physically active, independent activities are also good for their psychological and social growth. Children can develop self-awareness of autonomous activities, risk assessment insight, spatial awareness, wayfinding skills, and the ability to make decisions on their own, for example [6,7]. It allows children to cultivate exploration capabilities, communication skills, and environmental adaptability, as well as self-confidence, courage, seriousness, patience, a sense of responsibility, and a mindset of not being afraid of challenges [8].

The Chinese government has also paid attention to the issue, launching a series of guiding documents and including the construction of child-friendly cities as a pivotal point in the 14th Five-Year Plan to ensure children’s rights and spatial interests in the city and promote their independent activities. Nevertheless, most of the current research on CIAs in China focuses on street space and commuting space at the urban scale, while less on the residential level. As the primary space for CIAs, the quality of the spatial environment in urban residential areas has a decisive influence on children’s independent activities. Hence, the appropriate construction of outdoor spaces in residential areas is of critical significance to improve CIAs and promote children’s physical and mental health.

## 2. Literature Review

Methods such as questionnaires, interviews, children’s travel diaries, children’s cognitive maps, behavioral mapping, and GPS tracking methods are widely used in the study of permission for CIAs or the characteristics of CIAs [9,10,11,12,13,14,15]. Analysis and evaluation methods such as chi-square tests, regression analysis, principal component analysis, and the PRISMA system are frequently chosen by researchers in various countries [16,17,18,19,20,21]. Research has found that CIAs have declined significantly over the last 20 years, which has sparked extensive discussions in the fields of health, sociology, psychology, and geography [22,23,24,25,26,27]. Specifically, the intensity of children’s physical activity and factors influencing childhood obesity, children’s perceptions of the community and its safety, parental social attributes, social supervision and policy management were addressed.

The physical environment could be further split into the community environment, school environment, landscape environment, and traffic environment, and the study mainly focused on the impact of the commuting environment, traffic issues, walkability, infrastructure, and other related factors. A higher level of physical activity was found to be associated with accessibility and availability adjacent to greenspace, parklands, recreational areas, and sidewalks [28,29]. Research on the correlation between building density, walkability, traffic conditions, and CIAs in the community found that increased motorized traffic led to a reduction in CIAs [30,31,32,33,34]; Less traffic and more greenery helped to increase the proportion of CIAs [35,36]. Factors included in the walkability concept (such as land-use mix and residential density) have been shown to be crucial in relation to active commuting to school [37]. Holt even suggested that “low-walkable” neighborhoods (many cul-de-sacs) are more beneficial for younger children to get involved in outdoor play [38]. Riazi found that walkability was positively associated with girls’ independent mobility within a 400 m buffer, but not above an increasing distance [25]. In addition, Christine also declared that the size of the neighborhood influencing children’s physical activity was within a chosen distance of 500 m and 800 m buffers [27]. The above findings were informative for the study in selecting an appropriately scaled sample. It has also been demonstrated that well-designed neighborhood spaces and the availability of services for children’s needs had a positive impact on children’s early growth and development [39].

Social environment factors are mainly reflected in the regional economic situation, neighborhood safety, social supervision, and policy management [40,41,42,43]. Regarding individual-related factors, parental attributes [18,26,44,45], children’s characteristics [44,46,47], family economic level [21,26,48,49,50,51], and safety concerns [21,50] are included. Among them, personal perception of the built environment had a greater impact on children’s motivation to engage in activities. Parents’ safety concerns about strangers, gangs, and road traffic on the way to the place of play hindered their children’s opportunities for active and free play [52,53]. Edwards also indicated that parents’ perceived levels of neighborhood safety were linked to less conduct problems for kids aged 4 and 5 years [54].

Most studies have been conducted among children in developed countries, while studies in developing countries are still limited. The characteristic and quality of various types of neighborhood built environment can apparently differ. For instance, the paradigm of gated residential blocks, which refers to the enclosing of residential blocks by walls or fences, is applied by the majority of Chinese neighborhoods. The neighborhood employs closed management through access control, forbidding or preventing outside residents from approaching the interior. This kind of pattern was typical in China after 2000 for community construction, which was relatively more open in old urban blocks. Most of the neighborhoods in closed blocks adopted the layout of multi-story buildings, high-rise buildings, and a combination of both. And the public activity sites were mainly concentrated in the geometric center of the neighborhood, while few of them were open or decentralized at the entry.

The exploration of CIAs in China is still in its initial stage, focusing mainly on independent mobility and school commuting in the small area of the city region. Studies have revealed that traffic density and street interface in the community exert an impact on children’s independent activity [55]. Crowd activity, the number of stores and retail outlets, and the accessibility of schools and educational facilities are all positively associated with CIAs [56]. Individual characteristics, family attributions, and built environment factors influence children’s independent travel decisions [57]. Distance to activity sites, urban road networks, and functional mix degree are all factors that affect children’s independent travel [58]. However, all of these studies were biased toward children’s independent mobility in a 5–10 min range in the community for daily travel, focusing on various environmental factors such as transportation and land use along the route from home to destination, rather than on independent activities inside the residential neighborhood. The neighborhoods in this paper mainly referred to the unit residential block with boundaries defined.

Other studies in China have shown that factors such as site size, vegetation condition, diversity of activity facilities, environmental safety, and residential population affect children’s physical activity in residential outdoor spaces [59,60,61]. However, very few studies have explored the relationship between CIAs and these factors. It is clear that the factors involved in independent mobility and independent activity are not congruent, and further research should be conducted to explore the mechanisms of the impact for policies aimed at the improvement of the built environment to increase children’s independence and promote CIAs.

In general, a large number of studies have shown that the built environment has a significant impact on CIAs, and the available research provided a wealth of factors that might influence CIAs, which might partly intersect in different ways. However, the factors influencing CIAs may vary across national contexts and environmental factors. Therefore, further clarity is required about how outdoor space impacts CIAs in the Chinese residential neighborhoods. In contrast to the previous studies, we ask: are parents still sensitive and restricting children’s travel in the current stable social environment with very rare crime rates? Does the built environment have a similar impact on children’s independent mobility in open spaces of residential neighborhoods as in city streets? To address the research gaps and these questions, this paper proposed three main points based on the Chinese social and living contexts: (1) to explore the characteristics of CIAs and children’s preferences for independent activities in Chinese residential neighborhoods; (2) to assess the relevance of the built environment to CIAs in China; and (3) to evaluate the correlation between parents’ safety perceptions of the built environment, as well as their concerns about the permission for CIAs. Based on the presentation of the results, the study aimed to evaluate the influence of building environment factors on CIAs and the connection between parental decision-making and children’s permission for independent activities. It also sought to provide pertinent interventions that could serve as valuable references for CIAs promotion and children’s healthy development.

## 3. Materials and Methods

### 3.1. Study Methods

The study focused on children’s outdoor activity spaces in urban residential neighborhoods in Nanjing, China. Children’s activity spaces in three various types of residential areas, including high-rise, multi-story, and open residential areas, were selected as samples for the study through field research and classification. The characteristics and patterns of CIAs were summarized after observing and recording children’s independent outdoor activity behaviors.

Subsequently, a questionnaire survey was conducted to obtain guardians’ subjective evaluations of spatial safety elements, and a binary logistic regression model was established to analyze the factors influencing guardians’ decisions on children’s independent activities, considering the evaluation of residential environment elements and guardians’ guardianship style as independent variables, and whether children were allowed to move independently as dependent variables.

The built environment’s variables of outdoor activity spaces in neighborhoods were then subjected to quantitative analysis. The objective observation of the proportion of children’s independent activities was used as the dependent variable. The multiple built environment indicators were used as the independent variables to explore the correlation between the built environment and children’s independent activities through correlation analysis.

Finally, suggestions were presented to enhance the safety of children’s activity spaces in residential neighborhoods by fusing the distinctive patterns of CIAs and the components of outdoor activity spaces in residential neighborhoods.

### 3.2. Study Samples

**Figure 1:**
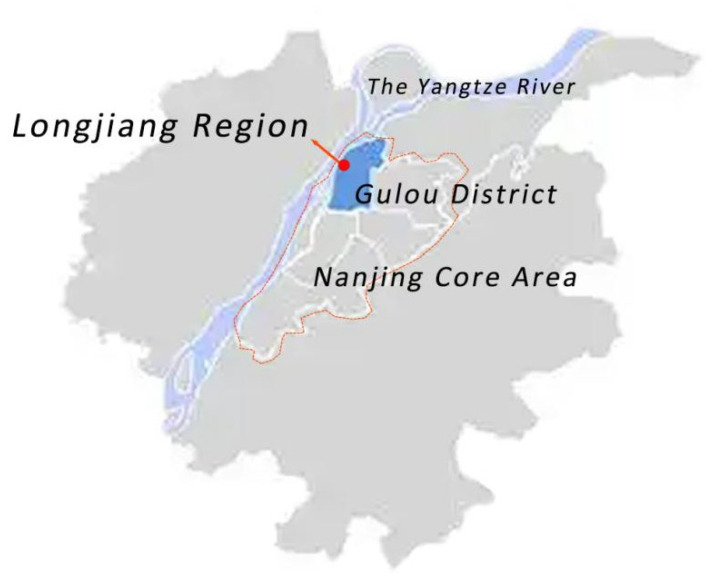
Location of Longjiang Region, Nanjing, China.

Nanjing, located in the east of China and the lower reaches of the Yangtze River, is a vital node city where the eastern coastal economic belt meets the Yangtze River economic belt strategically. It has long been one of the political, economic, and cultural centers in southern China. Gulou District is one of the core urban areas of Nanjing (Figure 1), with well-developed infrastructure, mature development, and many school-age children. Longjiang region is situated in the western portion of Gulou District, which was constructed and built in response to Nanjing’s rapid population boom in the 1990s. The area is mainly for residential functions, primarily in the form of enclosed residential neighborhoods, and is separated morphologically into multi-story, high-rise, multi-story, and high-rise combinations. The floor area ratio of residential areas is between 1.8 and 2.5, the road network density is 7.3 km/km^2^, the scale of the neighborhood is relatively well developed, and the residential areas and supporting construction are mature.

**Figure 2:**
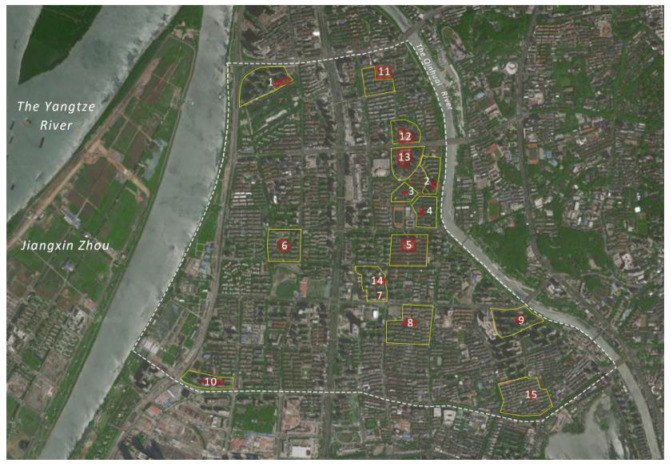
Locations of sample residential neighborhoods in Longjiang Region.

To reduce the interference of redundant factors outside the site, according to the conditions of residence closure degree, residential building form, location and size of the neighborhood, and degree of openness of the activity space, the following criteria were used to select the sample residential neighborhoods: (1) the land use of the public space for children’s activities was only for residence, not for urban green space and squares. (2) Within 500 m walking distance of the neighborhood was basically residential land with no urban or regional business and sports centers, and no commercial personnel or others interfered with children’s activities. (3) There were specific activity sites for local residents in the neighborhood, and the site served at least 500 households. Therefore, this paper selected 15 typical children’s outdoor activity spaces in the Longjiang Region as research objects, with 1–10 being children’s activity spaces in closed residential neighborhoods and 11–15 being children’s activity spaces in open ones. The following are the locations of the sample neighborhoods (Figure 2) and the current situation (Table 1).

### 3.3. Research Contents and Methods

#### 3.3.1. Behaviors of CIAs

The total number of CIAs and the type of independent activities in the sites were counted by methods of observation and behavior mapping. The survey was conducted via the stratified random sampling method, and two representative days of a school day and a weekend day with preferred and comfortable weather were selected to infer the general characteristics of children’s activities by probability. The research team carried out several pre-studies on the sample to learn about a basic understanding of the general situation of children’s activities under different climatic and weather conditions. Since Nanjing is a typical hot summer and cold winter city, the summer (June to August) is excessively hot, with temperatures between 30 °C and 39 °C and the winter temperatures (December to February) are typically between −11 to 2 °C and very humid. These are extremely unsuitable temperatures for outdoor activities and the number of independent activities among children is obviously low compared to the spring and autumn. For these reasons, the observation time were set in October, when the weather was favorable for children’s independent activities. During this time, students had just started a new semester, there was less study pressure, and more children had time to play outdoors. The research results were also meaningful for practice. Fifteen researchers with professional backgrounds conducted simultaneous observations on two sunny days—a weekday and a weekend day, in different sample residential neighborhoods from 9:30–11:30, 13:30–15:30, and 16:00–18:00. Children aged 0–12 years were categorized into preschoolers (0–5 years), lower elementary school children (6–8 years), and upper elementary school children (9–12 years) according to their independence competence. The number of children, independent activities and behaviors were recorded.

#### 3.3.2. Guardians’ Evaluation of CIAs and Residential Outdoor Space Safety

The impact of spatial safety on the neighborhood and children’s permission to engage in independent activities was investigated by a questionnaire. Studies indicated that the most important factors influencing guardians’ decisions on CIAs stem from concerns about the safety of children’s independent activities, which can be summarized as below: (1) concerns about neighborhood safety, including the level of neighborhood harmony and “stranger crisis” [49]; (2) concerns about the dangers of the community environment, including traffic, activity facilities, and activity spaces [50,62]; and (3) confidence and assurance of children’s independent activities [24,44], as evidenced by parents’ prior safety education and indirect supervision of children’s independent activities. However, most of the sample selected in this study were gated residential neighborhoods with strict access control systems and equipped with security booths and other facilities. Parents in the sample neighborhoods who were interviewed as part of the pre-study all acknowledged a high level of satisfaction with the neighborhoods’ harmony and safety, while factors (2) and (3) were more contentious. As a result, variables (2) and (3) were regarded as key considerations to enhance the relevance of the questionnaire. Finally, the questionnaire consisted of four sections: basic demographic information, children’s activity characteristics, safety events, and caregiving styles. Because children were not cognitively or expressively capable of filling out the questionnaire, the parental questionnaire was adopted. The pre-survey found that most of the children played with the elderly or nannies on weekdays, while parents were present for supervision on weekends. In order to ensure a reasonable distribution of age and crowd types of the respondents, the survey included data on a weekday and a weekend day.

#### 3.3.3. Objective Built Environment

Field measurements, software measurements, and qualitative analysis were used to obtain objective built environment data for the sample residential neighborhoods, including indicators at four levels: site, facilities, management, and the residential neighborhood.

### 3.4. Factors Influencing the Built Environment

Based on the literature review of existing studies, we summarized the relevant residential outdoor activity spatial elements and initially screened out the factors affecting children’s independent activities that appeared more frequently at four levels. Secondly, through field research, the current situation of the existing outdoor activity space in the sample settlements was analyzed, missing factors were added, and 17 influencing factors were finally determined as research variables, among which were eleven figure variables—site area; activity space area; density of entrances and exits; permeability coefficient of site boundary; ratio of tree coverage; ratio of shrub coverage; ratio of soft and hard paving; functional mix degree; density of seats; diversity of activity facilities; population density, and six classified variables—traffic safety; security; sanitation; facility safety; gated residential neighborhood; and building floors (Table 2).

## 4. Research Result

The investigation was conducted on a workday and a weekend day in mid-October 2021, when the weather was favorable for children’s outside activities. The majority of independently active children in the residential outdoor space were 6–12 years old, with the highest number of active children between 9:30–11:30 a.m. and 4:00–6:00 p.m., according to statistical data from 15 samples. 

### 4.1. Characteristics of CIAs

Spatially, the residential outdoor space was the major place for children’s daily activities. The frequency of children’s outdoor activities mainly ranged from 4–7 times per week, and the residential neighborhood was the main place for children’s activities. In terms of duration, 83.79% of children were active more than 3 times per week (4–5 times = 31.08%, 6–7 times = 34.80%, more than 7 times = 17.91%), and 77.03% of children spent more than 30 min per activity (0.5–1 h = 35.81%, 1–2 h = 38.18%, more than 2 h = 3.04%). Outdoor activities were the main way for children to do their daily activities (Table 3).

The demand for CIAs is directly proportional to age, with no discernible gender differences. The proportion of independently active children increased with age. 37.18% of active children aged 6–8 have independent activities, and the proportion of independent activities among children aged 9–12 is as high as 62.82%. With regard to gender difference, the independent activity of boys was slightly higher than that of girls, but there was more diversity among the sample settlements, such as 34.88% of girls were independently active in sample 6 and 100% in sample 11. After excluding the highest and lowest particular values, the statistics showed that more boys than girls were independently active in 8 samples, the same percentage of both sexes in 2 samples, and a smaller percentage of independent boys than girls in 3 samples (Table 4).

**Figure 3:**
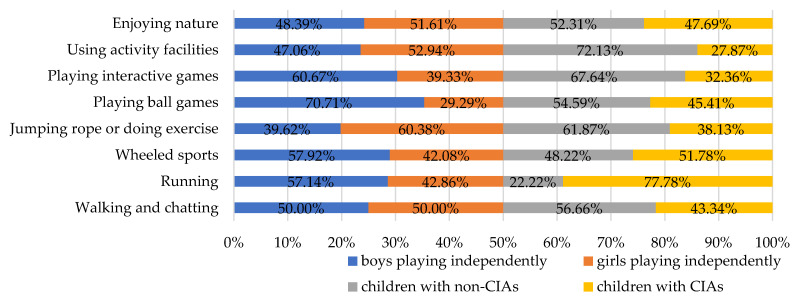
Gender difference of CIAs and proportion of CIAs and non-CIAs.

Children with independent activities had a wider range of activities than those who were not. The study discovered that independent children prefer path-based sports, such as bicycles, scooters, roller skating, and running, with 51.78% of independent children participating in wheeled sports and 77.78% of them running. The choice of activity type allowed them to move more widely and radiate throughout the site. Children who were not independently active were more likely to engage in activities such as enjoying nature, using activity facilities, playing interactive games, jumping rope, doing exercises, and taking a stroll. Children’s independent activity preferences were observed to vary by gender. Boys preferred energetic activities such as interactive games, ball games, wheeled sports, and running, accounting for 60.67%, 70.71%, 57.92%, and 57.14%, respectively. While independently active girls preferred relatively static activities such as being close to nature, active facilities, jumping rope and doing exercises, walking and chatting, and other less physically demanding activities (Figure 3).

### 4.2. Scenario Analysis of CIAs

Among the 15 sample spaces studied, six of the sample neighborhoods (classified as Type A neighborhoods) had a high proportion of CIAs, all above 40%. In contrast, the other nine sample neighborhoods (classified as Type B neighborhoods) had a proportion of CIAs below 30%. The differences of CIAs between the two types of neighborhoods were obvious. As a way to explore the relationship between the built environment factors of outdoor activities in neighborhoods and CIAs in terms of age, behavior, and range of activities, typical sites for CIAs in Type A neighborhoods were chosen to illustrate scenarios of CIAs in diverse spatial settings (Table 5).

#### 4.2.1. Subjects of Independent Activities

The majority of the independent outdoor activities are for children aged 6 to 12, and as they become older, the sort of outdoor activity evolves from low to high intensity. The younger children, around 6–8 years old, were fascinated by the outdoor spaces and mostly immersed in static activities. The activities they enjoyed included viewing plants, picking flowers and plants, collecting twigs and leaves, and playing with activity facilities. The independent activity preferences of 9–12 years old include running, jumping, riding, and other activities that were high-intensity and slightly challenging.

#### 4.2.2. Independent Activity Space

Independent activities for 6–8 years old children were those such as being near nature and using activity facilities. Activity facilities included exclusive play facilities for children and fitness facilities for adults in the neighborhood. The activity space depended upon the neighborhood’s functional space, and all the spaces were soft paving, such as lawn, sand pits, and plastic floors. Children aged 9 to 12 prefer ball sports or wheeled sports, and the space was primarily wide and hard-paved to adapt children to jogging and riding.

#### 4.2.3. Independent Activity Behaviors

Children’s independent activity behaviors are creative, but the ways and types of children’s activities were largely similar in different neighborhood spaces. Independently active children had established special “activity rules” in the outdoor spaces of current neighborhoods. They participated in treasure hunts by digging and picking plants, speed races on slopes and steps, as well as climbing activity facilities to view from the top.

#### 4.2.4. Range of Activity

The range of CIAs expanded with the increase of children’s activity intensity. Nature activities were centered on the landscape and radiated outward in a circle or fan shape. Facility-based activities took the facility site area as the boundary. In the open part of the courtyard, ball games formed an “invisible boundary,” and children played in groups. Cycling activities were provided in the form of curving paths and were not limited to a specific place.

### 4.3. Built Environment Differences of CIAs

A descriptive analysis of the built environment of Type A and Type B neighborhoods was carried out (Table 6) to investigate the reasons for the differences in the environments of the two types of neighborhoods as well as the reasons for the differences in the proportion of independent activities of children.

Site level. The area of the selected 15 sample sites ranged from 322 m^2^ to 15,960 m^2^, the activity space area ranged from 145 m^2^ to 2300 m^2^, the entrance and exit density ranged from 0.01 to 0.35, the interface permeability coefficient ranged from 0.32 to 0.849, and the shrub coverage ranged from 0.23–52.62%, the ratio of tree coverage ranged from 1.58–53.63%, the ratio of soft and hard paving was between 0–191.78%, and the functional mix degree was between 0–0.81. Among them, the average site area of Type A residential neighborhoods was 3238 m^2^, the average area of activity space was 721 m^2^, the density of entrances and exits was 0.06%, and the average functional mix around the site was 0.16, all of which were smaller than that of Type B residential neighborhoods. The average ratio of shrub coverage was 15.85%, the average ratio of tree coverage was 26.95%, and the average ratio of soft and hard paving was 42.7%, all of which were larger than that of Type B. In terms of interface permeability coefficient, the average values of indicators of the two types of residential neighborhoods were basically the same.

In terms of facility level, the density of seats in the sample residential neighborhoods ranged from 0.01% to 0.21% and the diversity of activity facilities ranged from 0 to 0.94. These two indicators in the Type A residential neighborhoods were higher than those in Type B, which were 0.66% and 0.22% higher respectively.

Management level. Residential neighborhoods with walkways around the site accounted for 33% of the total. 40% of the samples were equipped with security patrols. The proportion of samples with clean and well-equipped facilities was 87% and 67% respectively. Among them, Type A residential neighborhoods have stricter control over motor vehicles and better separation of people and vehicles. Furthermore, security, sanitation, and activity equipment maintenance were superior to Type B residential neighborhoods.

Residential neighborhood level. Gated residential neighborhoods made up 67% of the sample. The residential densities ranged from 2.71% to 8.50%, with high-rise neighborhoods accounting for 60% of the samples. Type A residential neighborhoods were all closed residential areas. Furthermore, in terms of building floors, multi-story and high-rise buildings in Type A residential neighborhoods were 17% less prevalent than in Type B residential neighborhoods.

Through a combination of subjective and objective analyses, the following is a more in-depth investigation of the influential relationships between elements of CIAs, the built environment, and guardians’ subjective evaluations in the fifteen samples of residential neighborhoods.

## 5. Relationship between CIAs and the Environment of Outdoor Activity Space in Residential Neighborhoods

### 5.1. The Influence of Guardians on CIAs Permissions

A total of 330 questionnaires were collected from the sample residential areas, and 296 valid questionnaires were obtained after sifting through them, with an effective rate of 89.7%. SPSS 21.0 was used to test the reliability and validity of the research questionnaire. The reliability of the questionnaire was expressed by Cronbach’s α-coefficient with a value of 0.821 (>0.8). The validity was tested by the KMO test and Bartlett’s test of sphericity, with a KMO value of 0.812 (>0.6) and a *p*-value of 0.000 (<0.01), indicating that the reliability and validity of the questionnaire were good and met the demand of statistical analysis.

In this study, the independent variables of the model are “Places where children’s safety incidents occurred” and “Ways to supervise children”. The dependent variable “whether children are allowed to move independently” was coded by 0 and 1, and a binary logistic regression model was used via SPSS21.0 to assess the impact of resident safety and the guardians on children’s independent activity decision (Table 7).

The finding reveals that waterfront areas in the neighborhood (95% CI 0.299, 1.113; *p* value = 0.100) and safety incidents at activity sites (95% CI 0.346, 1.091; *p* value = 0.097) reduced parents’ willingness to allow children to be independently active. Children who accepted safety education (95% CI 2.553, 7.142; *p* value = 0.000) and took mobile phones or positioning wristbands (95% CI 3.069, 15.71; *p* value = 0.000) were more likely to be permitted to play independently.

### 5.2. The Relationship between CIAs and Built Environmental Factors

To further clarify the relationship between CIAs and the built environment, the study took the proportion of children’s independent activities as the dependent variable and the built environment as the independent variable. SPSS 21.0 software was used to conduct a correlation analysis.

First, the numerical variables were tested for normal distribution, which was satisfied when the p-value was more significant than 0.1. Then, the Pearson correlation coefficient was used for the numerical variables that satisfied the normal distribution, and the Spearman correlation coefficient analysis was used for the numerical variables that did not satisfy the normal distribution. The independent sample t-test analysis was applied to the categorical variables, and the correlation coefficients and the significance of each index were finally obtained for the built environment and the proportion of CIAs (Table 8).

At the site level, the ratio of tree coverage had an association with the proportion of CIAs (*p* value = 0.023), with a correlation coefficient of 0.582. The proportion of soft and hard pavement was positively correlated with the proportion of CIAs (*p* value = 0.044), with a correlation coefficient of 0.527. A *p* value of 0.014 and a correlation coefficient of 0.617 showed a negative link between the functional mix of the neighborhood and CIAs. The proportion of CIAs was not significantly correlated with the size of the site, the size of the activity space, the density of entrances and exits, the permeability of the interface, or the shrub coverage.

There was a consistent trend at the facility level between activity facility diversity and the proportion of CIAs (*p* value = 0.033). The proportion of CIAs was not related to the density of seats.

At the environmental management level, sanitation status (*p* value = 0.020) was positively correlated with facility safety (*p* value = 0.013) and the proportion of CIAs, with correlation coefficients of 0.593 and 0.622, respectively. The correlation study found no correlation between traffic safety and security status.

At the residential neighborhood level, gated residential neighborhoods (*p* value = 0.005) and higher residential population density (*p* value = 0.030) favored CIAs. The residential building floors do not significantly affect the percentage of CIAs.

## 6. Discussion

### 6.1. Opportunities and Characteristics of CIAs

The WHO guidelines on physical activity and sedentary behavior, published in November 2020, stated that children should guarantee 60 min of moderate to vigorous aerobic activity per day, but only 41.22% of the children surveyed satisfied the requirements. This could be due to the fact that Chinese children’s outdoor activities are influenced by their guardians’ decisions, and their activity time is limited to some extent by their parents’ work schedules. The present study is in agreement with the finding that children’s activity hours during weekdays was remarkably shorter than during rest days due to parental availability on weekends [64]. According to interviews with Chinese parents, although social order has improved dramatically and they expressed high support for their children’s physical activity, they remained concerned about safety issues, which are particularly important for guardians. Consistent with some studies in developed countries, parental perceptions of the neighborhood-built environment may act as facilitators or barriers to young children’s play and interactions in their local environment [65]. Parents’ perceptions are an essential factor in children’s activities [66]. Besides, being addicted to electronic devices and affording a school workload may also contributing factors.

Furthermore, backyards in some nations provided plenty of areas for children to actively play to meet CIAs, but in others, such as Australia, smaller blocks and larger houses limit the space for children to play around [67]. In China, there is a comparable issue. Children’s access to a wide range of outdoor activities is constrained by the residential environments in China. Chinese families typically live in apartments instead of houses or villas, and in downtown Nanjing, for instance, the majority of the new residential areas are 18–33 story buildings, where children’s play areas are focused in public gardens inside the neighborhoods and their outdoor walking distance is significantly reduced, especially in high-density neighborhoods with lack of parks and squares around. These reasons limited children’s opportunities for outdoor play. All of the possible factors led to nearly 60% of children taking less than an hour of activity time in the questionnaires. The Trust for Public Land (TPL) of the USA launched the Schoolyards to Playgrounds program, which turned underutilized land resources into high-quality open spaces to alleviate a lack of public space, uneven distribution of urban green space, and inadequate community support for children’s development. TPL has renovated over 200 playgrounds in New York, grading and revitalizing vacant sites by upgrading sports and play facilities, resurfacing, and adding fencing [68]. This has resulted in a number of systematically linked activity spaces that encourage and promote independent activities for children in the neighborhood, as well as positive social responses. As for the limited urban living space in China, the layout of children’s activity space should be coordinated at the community level to enhance spatial and facility accessibility between neighborhoods. It is possible to renovate vacant land and transform it into an open ground for children’s outdoor play, personal growth and social interaction. Inter-neighborhood linkages should be strengthened to promote facility accessibility in order to build a harmonious neighborhood living circle and form a continuous, safe and diversified neighborhood children’s play network.

The following reasons may contribute to the age and gender differences in CIAs. Children naturally engage in independent activity, and as they grow, their self-awareness develops along with their mobility, increasing both their demand and the range of their independent play. Regarding gender, it is generally accepted that boys are physically stronger and possess more definite decision-making capabilities than girls [69]. Parents are therefore more inclined to give boys permission for independent activities at the same age. This was consistent with the finding of association between parental perceptions and independent mobility outcomes among children [70].

Based on the age-differentiated, gender-differentiated, and behaviorally creative characteristics of CIAs, different types of spaces should be created in the design of residential outdoor activity spaces to avoid spatial homogenization and homogeneity. For example, we could distinguish the activity space with different activity intensity and safety level according to age, and divide the cognitive space and game space in separate areas. Moreover, it is possible to combine the interactive space for each age group. And it is also important to distinguish the quiet and safe space for observation and exploration and the exciting or interesting space for adventure activities. Diana Memorial Playground in London, UK is a representative of British adventure playgrounds. It created “intentionally provided hazards” through climbing areas, sandpits and micro-terrain, to encourage children to challenge their physical and mental strength, and to cultivate their imagination and spirit of adventure [71]. This would be a meaningful approach to the design of residential neighborhoods in China. Sufficient square space needs to be reserved in advance to provide focused activities for children, such as ball games. Likewise, the design of the residential outdoor activity area necessitates clear movement zoning such as a continuous and safe bicycle traffic network to allow children to engage in independent activities such as cycling and jogging. In addition, the management of motor vehicles around children’s activity sites are supposed to be strengthened, and “buffer zones” should be set aside around the sites as appropriate to guarantee the safety and accessibility of children’s activity areas.

### 6.2. Built Environment and CIAs

#### 6.2.1. Site Level

The results showed that tree coverage was positively correlated with the proportion of CIAs. Trees can alter microclimate and help to improve thermal comfort in outdoor environments [72]. Multiple studies on greenery have shown that trees are naturally attractive to people and contribute to active physical activity [73,74]. The same was found to apply to independently active children in our research. Children were fond of being close to nature. With regard to tree coverage, trees usually do not obscure views and allow for good visual penetration of the site interior. Moreover, trees are also beneficial in improving neighborhood aesthetics and perceptions of safety and enhancing social cohesion [75,76]. Parents feel a natural affinity for such an environment and are more likely to allow for CIAs. For the analysis of the shrub coverage indicators, it was set based on security and visibility. Good sight penetration supports crowd surveillance [77] and a good shrub management can also improve regional image [78]. This can help to increase regional security and create a favorable environment for CIAs. Specifically, if the shrub covers below 1.5 m are large, children of short height may not be perceived by other people in the site when they play independently, and there is a risk of injury. The study put forth the hypothesis that shrubs below 1.5 m would lessen sight penetration and site safety resulting in poor CIAs. However, the correlation analysis did not reveal any relationship between shrub cover and CIAs. This might be because visibility was unaffected by the shrubs since they were largely at the edge of the sample activity sites. Moreover, interviews revealed that most site users did not noticeably perceive the interface permeability coefficient and shrub cover. The above reasons might account for the lack of significant correlation in this metric. However, at the site design level, greenery is still an important site design indicator. In addition to the aesthetic and experiential qualities that come with greenery, guidelines for the Construction of Child- Friendly Communities in Shenzhen, China, suggests that trees with tall canopies can be placed in the site to meet shade needs, and shrubs below 0.8 m can be placed at the boundary of the site to divide the area and isolate the role of motor vehicles [79]. Some greenery can also be used to prevent mosquitoes and repel insects. Above all, the openness of trees and the enclosing nature of shrubs play an important role in ensuring the safety of children’s activities.

Contrary to expectations, the high functional mix around the activity site is not conducive to CIAs. This indicator was based on existing research on children’s activity preferences, some of which showed a positive correlation between functional mixing and children’s physical activity [25,26]. The analysis of the built environment of the study sample revealed the following possible reasons for such results. In neighborhoods with only function for residence, the population tends to be homogeneous and fixed. Guardians and children may gradually relax vigilance in such an environment. Conversely, some of the activity sites are surrounded by kindergartens, community centers, property centers, kiosks, and courier points. The diverse functions were often accompanied by a complex crowd, such as parents who took their children to and from school, commercial shoppers, and office workers of community centers. The various identities of people moving around the sites raised parents’ concerns about children’s safety, which in turn led to a decrease in the proportion of CIAs.

#### 6.2.2. Facility Level

The findings also indicated that CIAs are promoted by a variety of activity facilities, which enriched different kinds of activities available to children and stimulated interest in and motivation for the activities. Furthermore, a wide variety of activity facilities can satisfy the diverse requirements of the residents, attracting people of all ages and thereby raising the possibility of resident surveillance responsibilities, which in turn lowers the sense of risk to users and promotes CIAs. To that end, the kind and number of facilities should be evaluated concerning the condition of different neighborhoods, and recreational facilities and leisure facilities are supposed to be paid attention to in terms of quality, materials, signage, colors, and so on.

Additionally, it was discovered during the interviews that the barrier-free facilities of the activity site could improve the accessibility of younger children and the safety of activities in the site, as well as exercise the possibility of their independent activities, given that the majority of outdoor activities for infants and toddlers would be assisted by strollers and three-wheeled bicycles. For facility maintenance, the neighborhood should build a long-term facility supervision and maintenance mechanism, encourage parents and children to engage in the collaborative construction and governance of activity spaces, and create appropriate sites for children to relax and recreate. Based on the characteristics of CIAs for different age groups, it is preferable to build a more precise index system of community facilities for children along with the allocation of diverse facilities. It’s also vital to consider about how to support and provide age-mixed and dynamic community facilities and space configurations.

The open space, sports & recreation strategy developed by Wokingham Borough Council in the UK set out the standards and minimum size of facilities for children’s activities in residential communities, such as a minimum of 100 m^2^ of neighborhood-scale children’s activity space. The council also introduced play space design guide, which suggests different play typologies of open space proposed for all kinds of groups of children on this basis. Particularly it addresses the inclusive play space that will allow children, young people and adults of all ages to interact together and with their environment—a place where a diverse range of users can play in a variety of ways and learn from one another [80]. Shanghai Dinghai Community has achieved a social climate of the intergenerational integration, through dynamic and exploratory facilities and spaces such as the “Community Garden Renovation”, “Stray Cat House” and “Secondhand Bookstore”. Such a strategy promotes children’s independent exploration and communication, which aids in the development of their social and self-care competencies.

#### 6.2.3. Management Level

The study also found that users’ perceptions of the environment largely influenced their behavioral activities and that guardians’ perceptions of the safety of outdoor spaces influenced their decisions about children’s independent activities. At the management level, sanitation and facility safety can affect CIAs. It is recommended that activity sites are supposed to be built in residential areas with clear sightlines and more sight-reachable places based on parents’ and children’s perceptions of environmental safety. Furthermore, we can improve monitoring and management by placing security booths around activity sites, configuring the camera, and assigning security guards to patrol and maintain site sanitation. These can improve guardians’ sense of security for children’s outdoor spaces and promote CIAs by increasing “residential awareness”. In terms of facility maintenance, we propose a long-term facility supervision and maintenance mechanism, which can encourage parents and children to participate in the joint construction and governance of activity places in order to create places that cater to children’s demands for rest and play. Practice in Changsha, China, has shown that establishing children’s councils and involving parents and children in neighborhood planning, implementation, and construction could well improve their understanding and trust in the social environment and increase their sense of “ownership” of the environment. This prototype of resident-led shared management is critical for increasing parents’ and children’s sense of environmental security and promoting parents’ permission for CIAs.

#### 6.2.4. Residential Neighborhood Level

The study showed that the closeness of the neighborhood had a positive effect on CIAs, which was in line with the findings in Western countries that lower street connectivity increased the likelihood of children being active [81]. Street closure interventions might be effective in increasing children’s physical activity [38]. Children did seem to be more independent in their activities when living in a relatively enclosed residential environment, which would increase parents’ and children’s safety perception. Likewise, studies conducted at larger community scales have revealed a connection between children’s outdoor activity and traffic safety, with the presence of crosswalks or traffic lights being positively correlated with outdoor play [82]. Nevertheless, we discovered that the sidewalk outside the site had no discernible impact on the CIAs at a smaller neighborhood scale. This might be a difference due to the size of the traffic volume, and even though the children’s activity site was close to a driveway, the frequency of vehicle presence was much smaller compared to urban roads, potentially negating the traffic effect. Therefore, in the practice of community design, it is necessary to improves continuous paths and expand the exploration space for children that can be safely accessible in the closed neighborhood. Organic linkage of residential inter-building activity space, neighborhood outdoor activity space, supporting services, and walking paths is highly recommended to establish a children’s slow walking system. Chengdu Cuqiao subdistrict of China, for example, established a children’s slow passage with cartoon elements, as well as slow signs and speed bumps at children’s crossing entrances to string together children’s activity facilities in the community in order to protect children’s traffic rights and created safe activity paths. Traffic order optimization can improve the quality and safety of children’s activity spaces, thereby improving CIAs.

A previous study on youth in Nanjing found a consistent and graduated association between residential density and physical activity, those living in higher-density areas being less physically active [83]. As for children, our study found that neighborhoods with higher residential densities tend to incentivize independent activities among children. It is possible that in a more densely populated neighborhood, there are more active people engaging in daily outdoor activities. A relatively fixed population is conducive to the establishment of “familiar neighborhoods” and the promotion of harmony and friendliness, where parents are more comfortable with children being alone in the neighborhood. Neighborhoods of higher residential densities may have more school-age children and they will have more playmates, which may also contribute to the increase in CIAs. In addition, it has been shown that users’ perceptions of environmental safety largely influence their behavioral activities. More soft paving, good sanitation, secure activity facilities, and strict access control can enhance occupants’ psychological comfort and sense of security, build trust in the outdoor activity space and reduce the sense of stranger crisis [84].

Site area, activity space area, and density of seats may influence children’s behavior but have less impact on the proportion of CIAs. Due to the centralized layout of activity spaces inside Chinese neighborhoods, and the clear domain boundaries between residential blocks, it is rare for children within a neighborhood to play alone in another one. When the possibility of self-selection of play space was reduced, the site area, activity space area, and seating density appeared to become “unselectable variables”, and the study failed to investigate the significant correlation between these indicators and CIAs in the 15 samples. Perhaps in more open spaces or urban public spaces, these variables would have an impact. The survey also discovered that children with independent ability are very familiar with elevator use. The time consumed for children living in high-rise and multi-story residential areas travelling to the activity space is both typically less than 5 min, and the convenience is not much different. As a result, the link between building floors and CIAs was tenuous.

### 6.3. Limitations

The number of samples was modest because it only included the residential parts of Longjiang Region in Nanjing, which may have constrained the findings of the analysis. Additionally, objective data were obtained from on-site observations, and the study only collected information on children playing in the fall. However, children’s activities may vary depending on the weather or other environmental factors. Whenever possible, the characteristics of children’s activities in each season and under different weather conditions should be fully considered to further clarify the characteristics of CIAs under different climatic and weather conditions and their relevance to the built environment of the neighborhood. The interview-based questionnaire is relatively subjective. Although some parents consulted with their children, there might still be cases where the children’s real thoughts were ignored.

Future studies are expected to broaden the coverage of the sample and increase the number of observation periods for CIAs. Children’s discussion groups could also be supplemented to acquire more perspectives. These would help to increase the accuracy and generalization.

## 7. Conclusions

This paper discussed the issue in the context of the construction of child-friendly cities. The relationship between objective built environment factors and CIAs was empirically analyzed and explored in residential neighborhoods, and it was beneficial to fill up relative gaps in previous research on CIAs at the residential neighborhood level in China. With regard to the current neighborhood construction in China, the pattern of enclosed and managed residential neighborhoods with clear neighborhood boundaries and certain open spaces for children’s activities has led to a tendency for CIAs to be concentrated in the internal areas of neighborhoods. Children’s behavior mapping and questionnaires revealed that CIAs are required by children of all ages. CIAs are also characterized by creativity in behavior and excitement in games. Overall, the study discovered that the built environment factors in the samples had a strong influence on CIAs, including tree coverage, functional mix degree, facility safety, gated neighborhoods and population density. In addition, we also found that factors affecting CIAs were not entirely consistent with those promoting physical activity. Besides, parents’ perception of the safe neighborhood built environment was especially crucial for CIAs. Parents have expressed great concern about the waterfront areas in their neighborhoods, so children’s activity areas should be kept as far away from the waterside as possible, and protection measures for the dikes should be strengthened.

From our study, we could realize that the built environment factors in residential neighborhoods affect CIAs in different forms. It is essential to stimulate children’s motivation toward vigorous activities in order to encourage them to participate in more physical activities, whereas rather more importantly for promoting CIAs is the enhancement of residents’ perceptions of the safety of the residential children’s activity space. The indicators of satisfying tree coverage, a neat and sanitary neighborhood environment, and a higher proportion of soft paving increased the likelihood of CIAs through visual perception. Some indicators, on the other hand, might have an indirect impact on CIAs, such as a lower functional mix and much more enclosed residences, which would limit crowd mobility and reduce site disturbance by unrelated people. In addition, densely populated residential areas promoted CIAs by increasing users’ perception of site safety through “Street eye” or by raising “neighborhood awareness”. In conclusion, we should raise awareness about the provision for children’s activity sites in neighborhoods, as well as make appropriate improvements to the built environment in light of the primary demand of parents or children, in order to improve the comfort, safety, and durability of the sites and thus promote CIAs. Local government departments should exert their roles to explore a more systematic and precise policy system, as well as construction guidelines that meet the needs of local residents with regard to the features of urban development and community types, in order to provide a suitable reference for the construction of child-friendly cities.

The method and result of this study have practical implications for identifying the characteristics of CIAs and exploring the factors influencing CIAs at the residential neighborhood level in China. Future research needs more observation time and wider or more precise classifications of factors, such as the impact of residential neighborhoods in different regions and seasonal changes on CIAs. This research will provide practical child-friendly adaptations to existing neighborhoods and design recommendations for future planning to improve CIAs and promote the health and well-being development of young children.

## Figures and Tables

**Table 1:** Pictures of the sample residential neighborhoods.

Number	1	2	3	4	5
Status	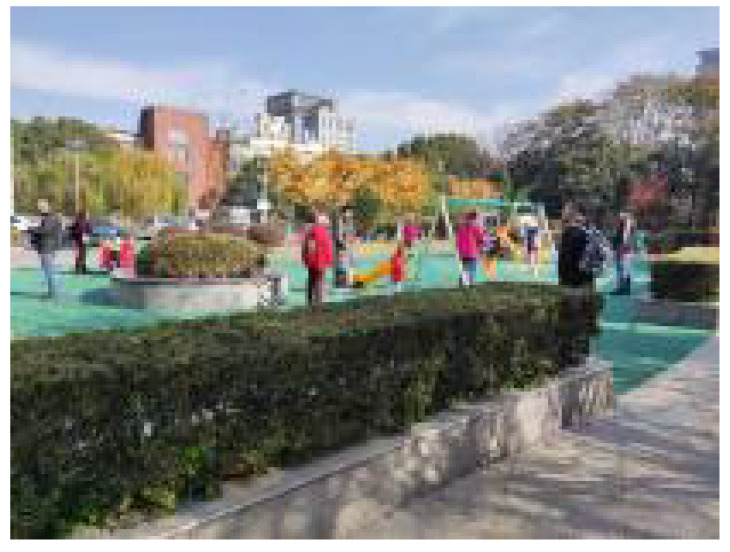	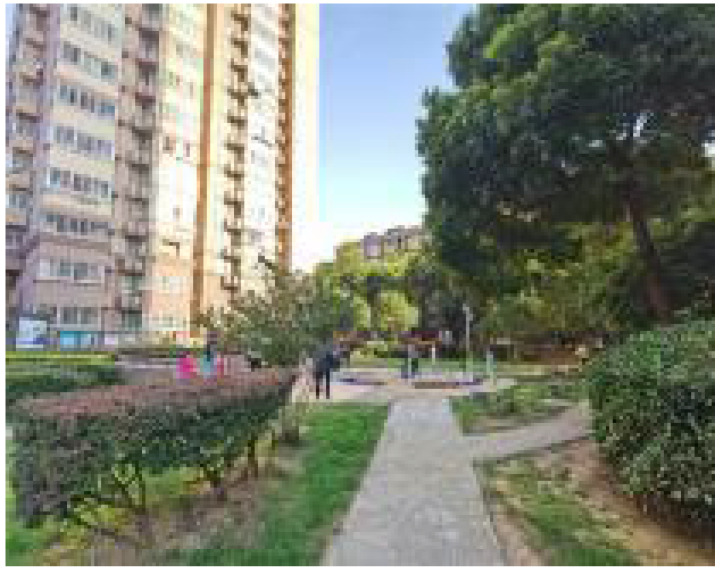	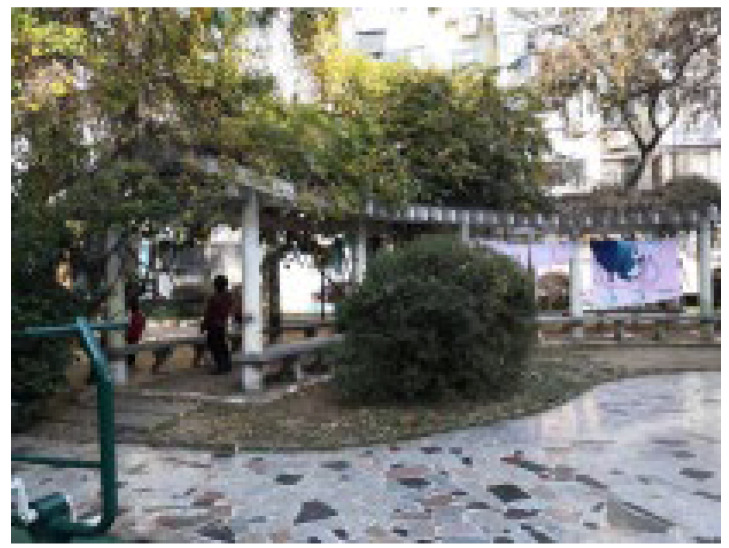	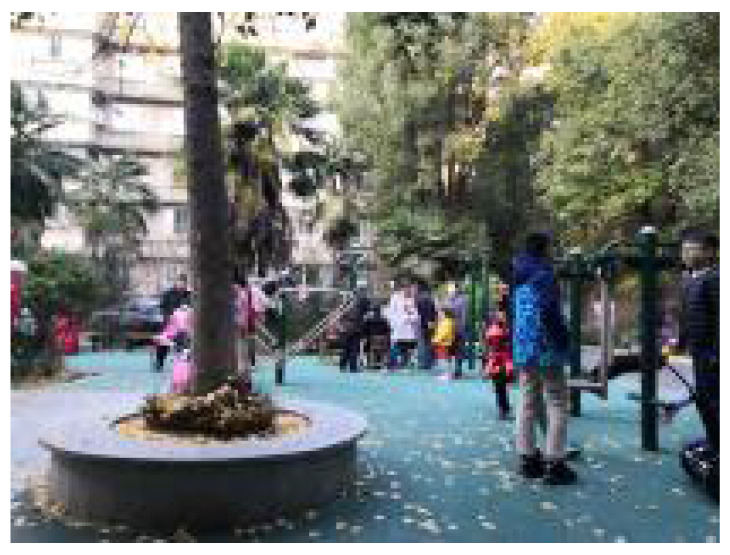	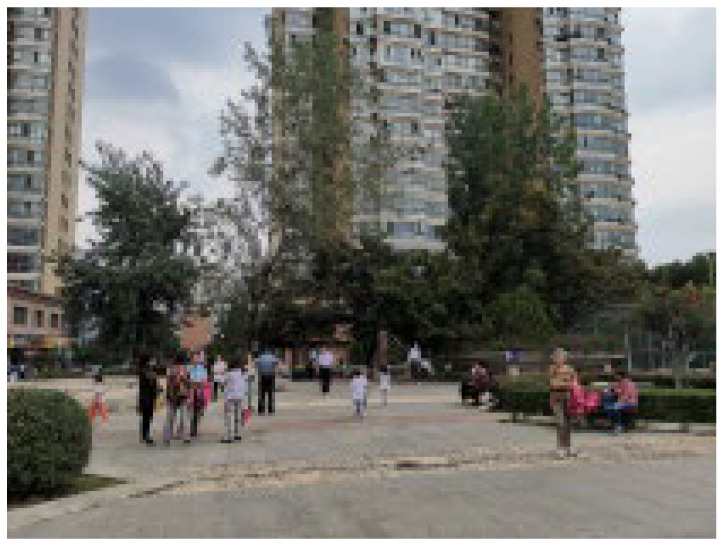
**Number**	**6**	**7**	**8**	**9**	**10**
Status	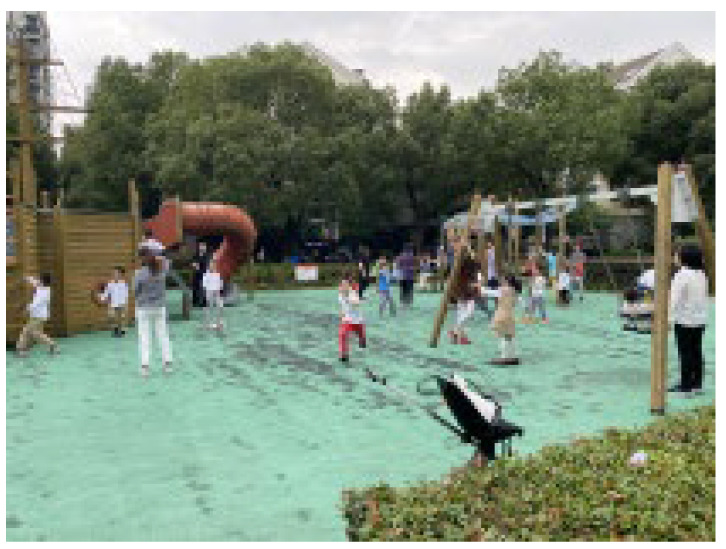	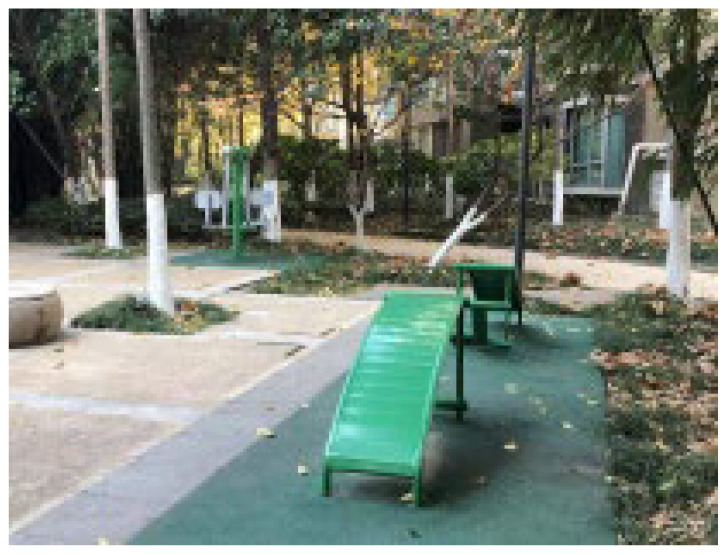	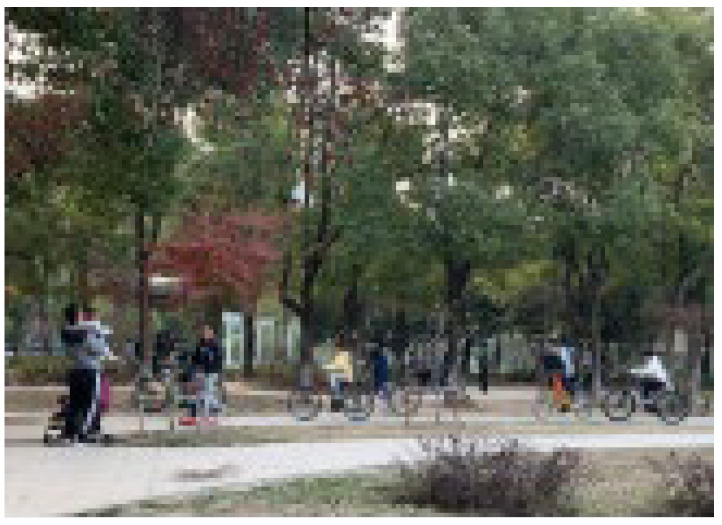	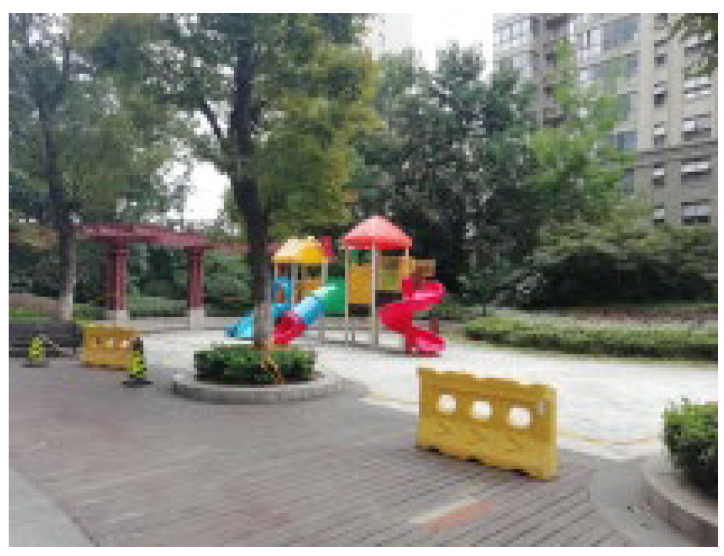	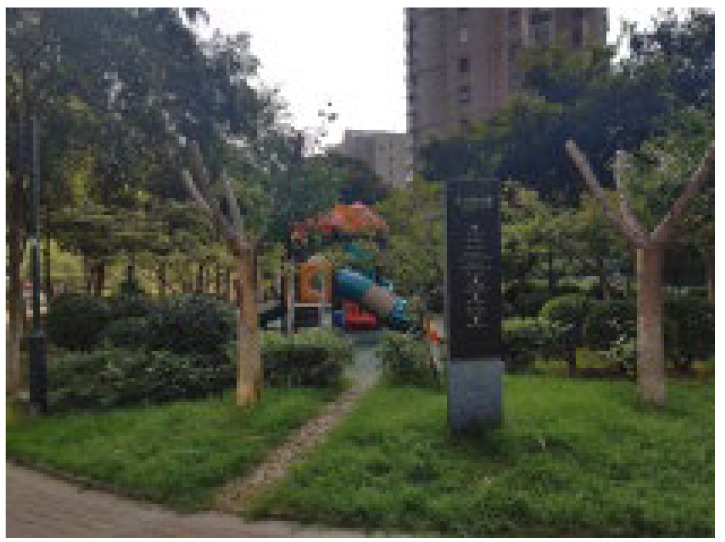
**Number**	**11**	**12**	**13**	**14**	**15**
Status	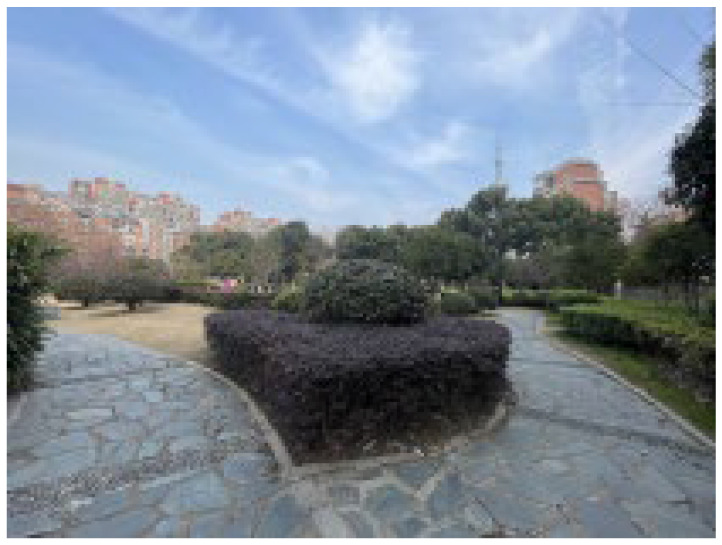	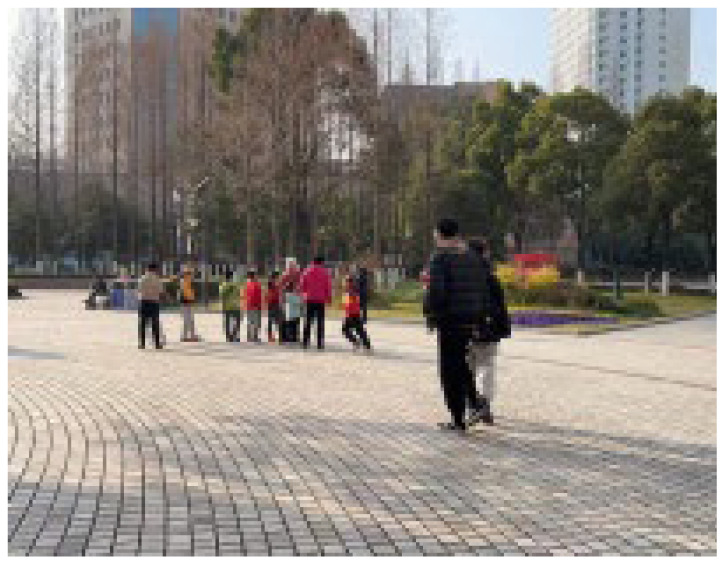	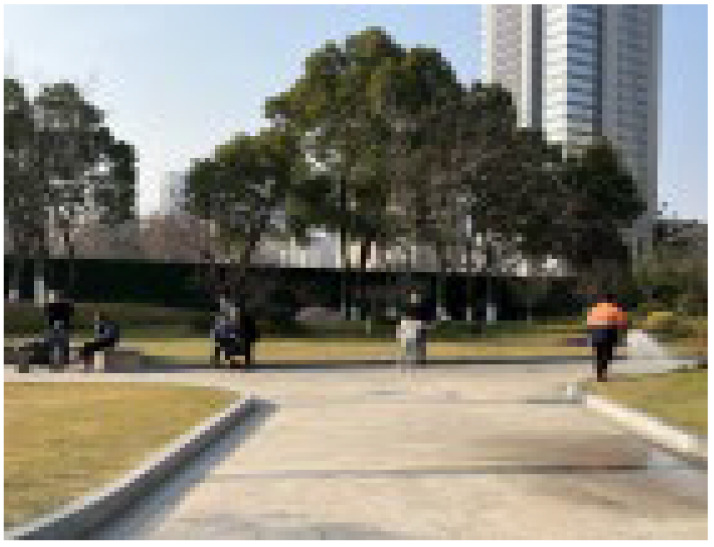	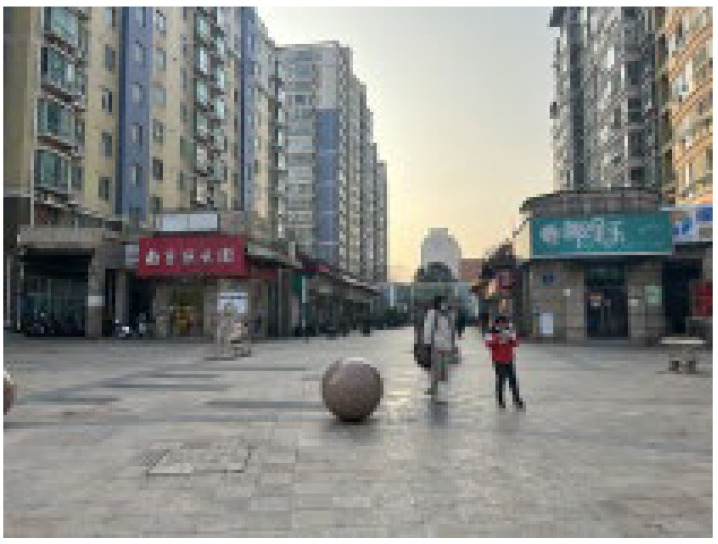	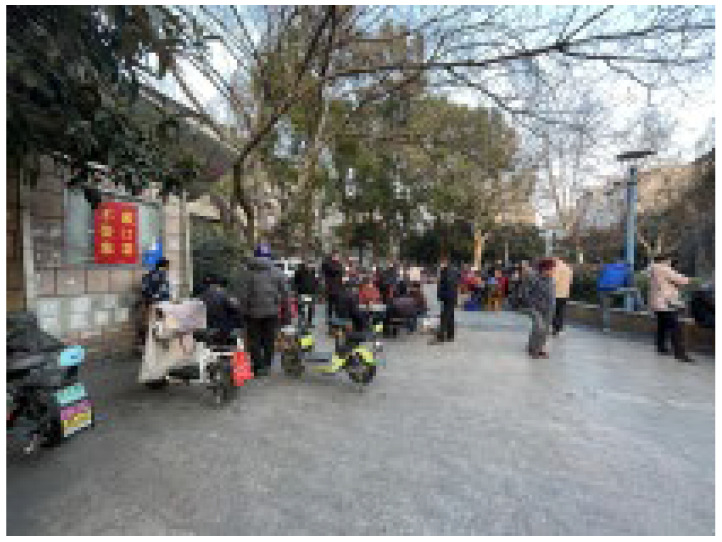

**Table 2:** Definition and calculation formula of built environment factors.

Category	Variable	Definition	Calculation Formula	
Site Level	site area	Area of the activity space.	Measured via CAD Field research and measured via CAD	
	activity space area	Area of the activity space available for CIAs.		
	density of entrances and exits	Ratio of the total length of the entrance and exit of the site to the perimeter of the site boundary.	$D_{c}=\frac{L_{c}}{C_{i}}$	$\mathrm{Where}\;L_{c}$$\;\mathrm{is}\;\mathrm{the}\;\mathrm{total}\;\mathrm{length}\;\mathrm{of}\;\mathrm{the}\;\mathrm{entrance}\;\mathrm{and}\;\mathrm{exit}\;\mathrm{of}\;\mathrm{site}\;i;\;C_{i}$ is the perimeter of site i.
	permeability coefficient of site boundary	The degree of closure and openness of the site boundary.	${\;P}_{i}=\frac{0\times a_{1}+0.5\times a_{2}+1\times a_{3}}{C_{i}}$	$\mathrm{Where}\;a_{1}$$\;\mathrm{is}\;\mathrm{the}\;\mathrm{total}\;\mathrm{length}\;\mathrm{of}\;\mathrm{closed}\;\mathrm{interface}\;\mathrm{of}\;\mathrm{site}\;i;\;a_{2}$$\;\mathrm{is}\;\mathrm{the}\;\mathrm{total}\;\mathrm{length}\;\mathrm{of}\;\mathrm{semi}-\mathrm{closed}\;\mathrm{interface}\;\mathrm{of}\;\mathrm{site}\;i;\;a_{3}$$\;\mathrm{is}\;\mathrm{the}\;\mathrm{total}\;\mathrm{length}\;\mathrm{of}\;\mathrm{open}\;\mathrm{interface}\;\mathrm{of}\;\mathrm{site}\;i;\;C_{i}$ is the perimeter of site i.
	rate of tree coverage	The ratio of shadow area by vertical projection of trees to the site area.	$S_{q}=\frac{S_{t}}{S}$	$\mathrm{Where}\;S_{t}$ is the shadow area shadowed by vertical projection of trees; S is the area of site i.
	rate of shrub coverage	The ratio of shadow area by vertical projection of shrubs below 1.5 m to the site area.	$S_{g}=\frac{S_{y}}{S}$	$\mathrm{Where}\;S_{y}$ is the shadow area shadowed by vertical projection of shrubs below 1.5 m; S is the area of site i.
	rate of soft and hard paving	The ratio of the area of soft paving (e.g., plastic) to hard paving (e.g., granite) in the site.	$S_{p}=\frac{S_{r}}{S_{y}}$	$\mathrm{Where}\;S_{r}$$\;\mathrm{is}\;\mathrm{the}\;\mathrm{area}\;\mathrm{of}\;\mathrm{soft}\;\mathrm{paving};\;S_{y}$ is the area of hard paving.
	functional mix degree	The average value of information entropy of functional facilities around the site.	$F_{s}=-\overset{N}{\underset{i=1}{\sum }}P_{i}\times \log P_{i}\;(P_{i}=\frac{A_{i}}{\sum_{i=1}^{N}A_{i}})$	$\mathrm{Where}\;P_{i}$$\;\mathrm{is}\;\mathrm{the}\;\mathrm{probability}\;\mathrm{of}\;\mathrm{information}\;\mathrm{entropy}\;\mathrm{for}\;\mathrm{class}\;i\;\mathrm{facilities};\;A_{i}$ is the number of class i facilities.
Facility Level	density of seats	Ratio of the number of seats in the site to the site area.	$D_{z}=\frac{N_{z}}{S_{i}}$	$\mathrm{Where}\;N_{z}$$\;\mathrm{is}\;\mathrm{the}\;\mathrm{number}\;\mathrm{of}\;\mathrm{seats}\;\mathrm{in}\;\mathrm{site}\;i;\;S_{i}$ is the area of site i.
	diversity of activity facilities	The Simpson index is used for reference [63]. The larger the value, the more abundant the types of facilities and the more uniform the distribution of facilities with different functions.	$D_{m}=1-\overset{n}{\underset{i=1}{\sum }}{\left(\frac{N_{i}}{N}\right)}^{2}$	$\mathrm{Where}\;N_{i}$ is the number of activity facility type i in the outdoor activity space; N is the total number of activity facilities; n is the total number of types of activity facilities in the outdoor space.
Management Level	traffic safety	The periphery of the site is the walkway.	YES = 1; NO = 0	
	security	The residential area is patrolled by security guards.		
	sanitation	The site is clean and tidy.		
	facility safety	The activity facilities in the site are of good quality and undamaged.		
Residential Neighborhood Level	gated residential neighborhood	The site is in the gated residential neighborhood.	YES = 1; NO = 0	
	population density	The ratio of the number of residents in the radiation radius of the site to the total radiation area. (2.62 pers/household according to the 2021 census)	$D_{i}=\frac{2.62\times N_{i}}{S_{i}}$	$\mathrm{Where}\;N_{i}$$\;\mathrm{is}\;\mathrm{the}\;\mathrm{total}\;\mathrm{number}\;\mathrm{of}\;\mathrm{households}\;\mathrm{living}\;\mathrm{within}\;\mathrm{the}\;\mathrm{radius}\;\mathrm{of}\;\mathrm{the}\;\mathrm{site}\;i;\;S_{i}$ is the radiated area of site i.
	building floors	High-rise buildings or multi-story buildings in neighborhoods.	High-rise buildings = 1; multi-story buildings = 0	

**Table 3:** Statistics on the characteristics of CIAs.

Category	Variable	Frequency	Percentage (%)
frequency of activities	6–7 times	103	34.80
	4–5 times	92	31.08
	more than 7 times	53	17.91
	2–3 times	42	14.19
	less than 1 time	6	2.03
duration of activities	1–2 h	113	38.18
	0.5–1 h	106	35.81
	15–30 min	62	20.95
	more than 2 h	9	3.04
	less than 15 min	6	2.03
places of activities (Multiple Choice Questions)	children-only activity space	276	34.12
	square in the residential neighborhood	233	28.80
	school playground	121	14.96
	park	83	10.26
	open space in the front of the unit building	46	5.69
	commercial entertainment venue	27	3.34
	others	43	5.32

**Table 4:** Statistics on the age and gender differences of CIAs in sample neighborhoods.

Category	Variable	Frequency		Percentage (%)	
age	9−12 years old	468		62.82	
	6−8 years old	277		37.18	
number	number and ratio of gender in each sample residential neighborhood of CIAs	boy	girl	boy	girl
1		14	14	50.00	50.00
2		47	46	50.54	49.46
3		21	16	56.76	43.24
4		48	32	60.00	40.00
5		28	20	58.33	41.67
6		84	45	65.12	34.88
7		3	6	33.33	66.67
8		89	56	61.38	38.62
9		13	9	59.09	40.91
10		17	11	60.71	39.29
11		0	2	0.00	100.00
12		25	30	45.45	54.55
13		12	32	27.27	72.73
14		10	8	55.56	44.44
15		3	3	50.00	50.00

**Table 5:** Scenario analysis of CIAs.

Activity Behavior	Diagram of the Site	Photos	Scenario Analysis of CIAs
Enjoying nature	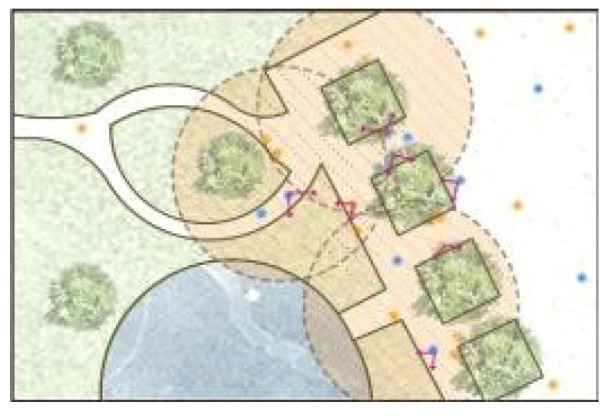	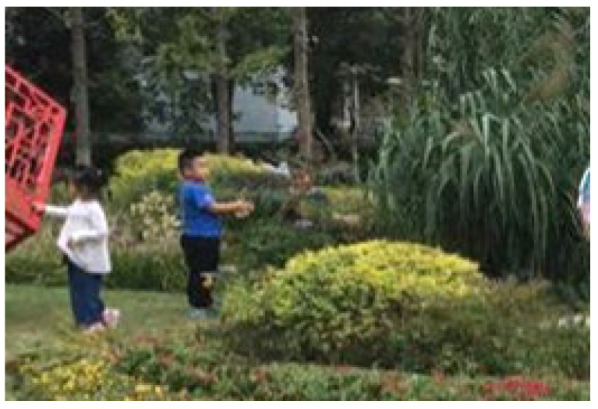	Children’s age: 6–8 years old Activity Behavior: observing plants, picking flowers and plants, collecting twigs and leaves Range of Activity: radiating outward from the center of the landscape with a circular or fan shape
	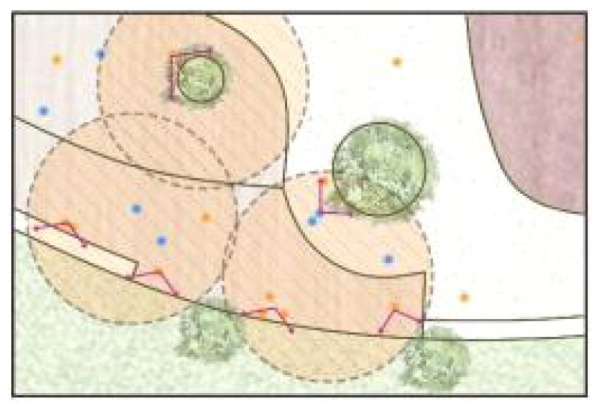	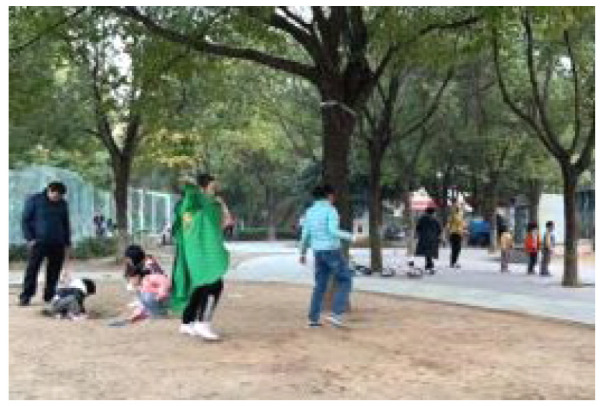	
Using activity facilities	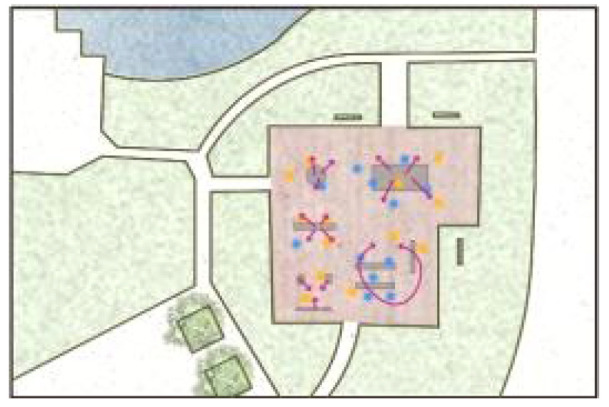	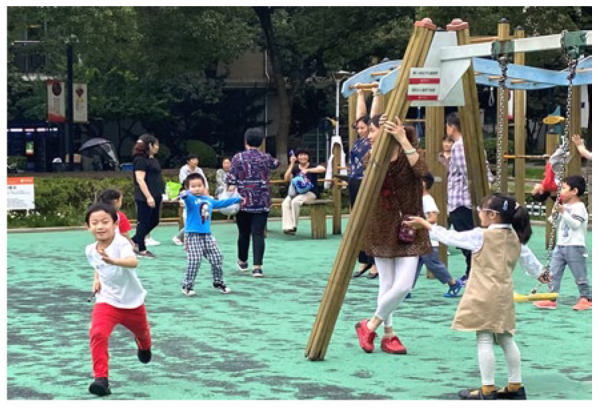	Children’s age: 6–8 years old Activity Behavior: chasing, climbing, playing activity facilities Range of Activity: radiating outward from the center of large facilities or concentrating inwards around group facilities
	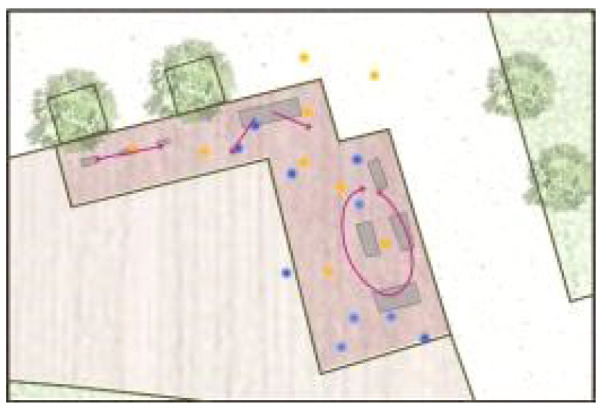	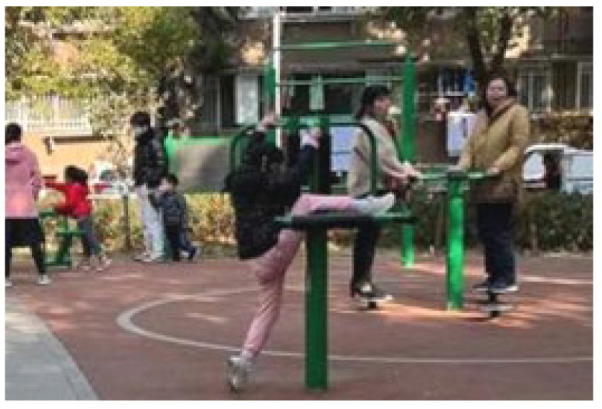	
Playing ball games	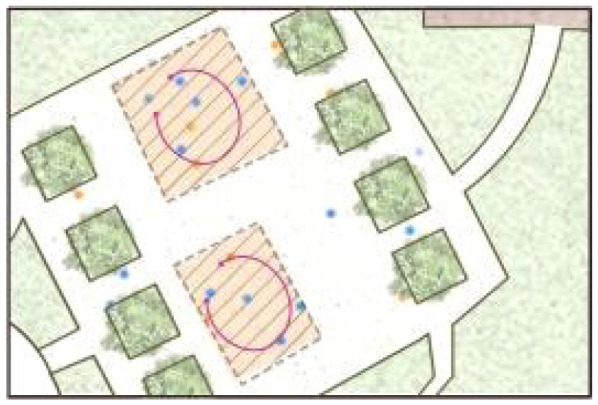	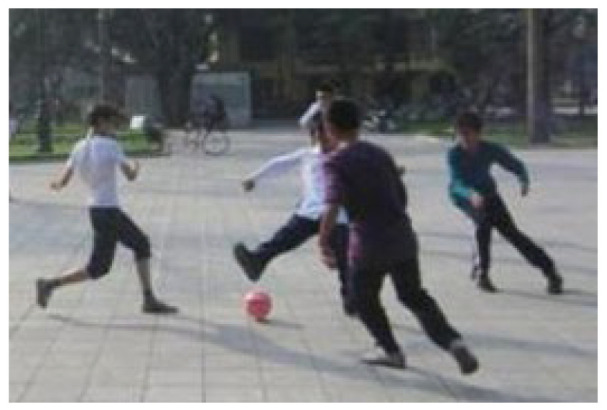	Children’s age: 9–12 years old Activity Behavior: Kicking ball, playing “goalkeeper” game, badminton and volleyball Range of Activity: in the open area of the square within an “invisible” boundary
	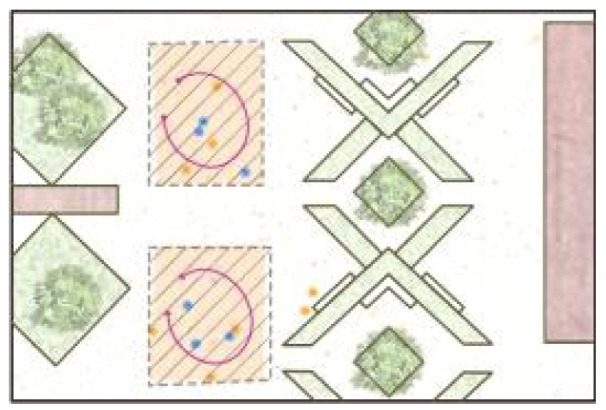	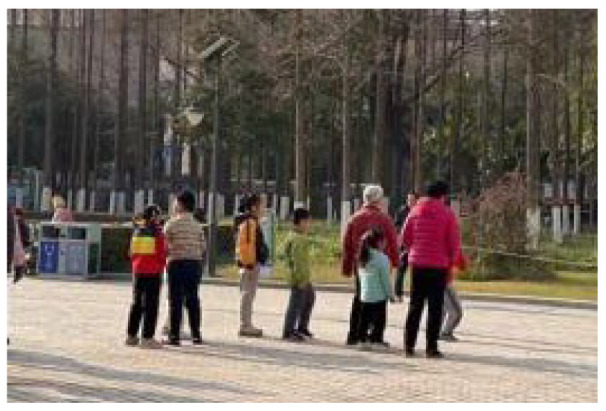	
Wheeled sports	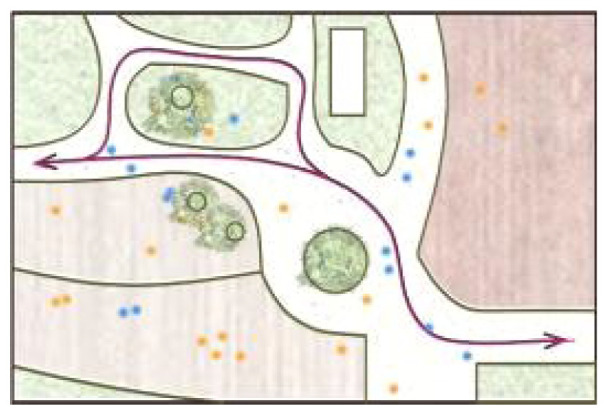	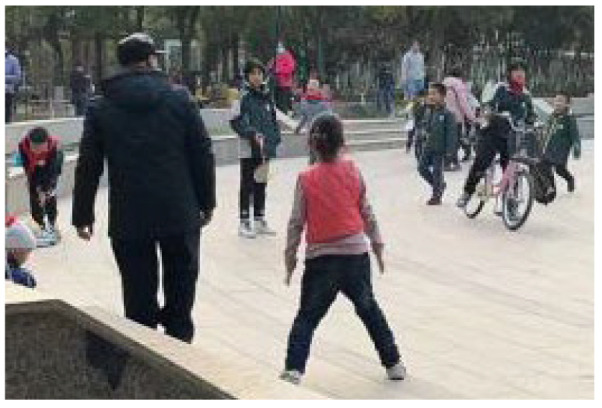	Children’s age: 9–12 years old Activity Behavior: cycling, rollerblading, skateboarding, riding balance bikes, etc. Range of Activity: a tendency to move freely with a relatively fixed path
	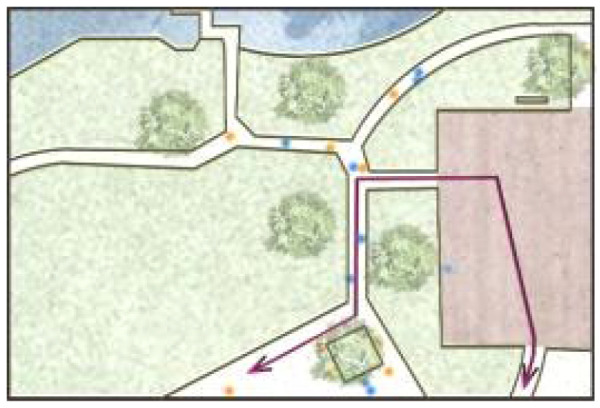	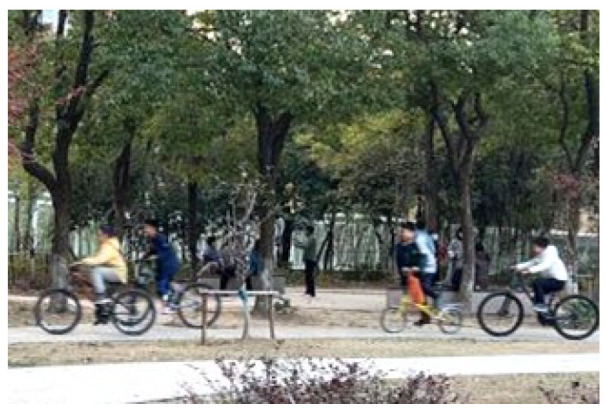	

**Table 6:** Built environment and proportion of CIAs in sample residential neighborhoods.

Category	Variable	Children Active Independently as a Percentage of Total Children > 40%		
		Yes (Type A)	NO (Type B)	In Total
		Average Value	Average Value	Average Value
Site Level	site area	3238 m^2^	7422 m^2^	5748 m^2^
	activity apace area	721 m^2^	1537 m^2^	1211 m^2^
	density of entrances and exits	0.06%	0.11%	0.09%
	permeability coefficient of site boundary	0.49	0.51	0.50
	ratio of tree coverage	26.95%	7.53%	15.30%
	ratio of shrub coverage	15.85%	10.78%	12.81%
	ratio of soft and hard paving	42.70%	33.67%	37.28%
	functional mix degree	0.16%	0.51%	0.37%
Facility Level	density of seats	0.91%	0.25%	0.52%
	diversity of activity facilities	0.88	0.66	0.75
Management Level	traffic safety	50%	22%	33%
	security	50%	33%	40%
	sanitation	100%	78%	87%
	facility safety	100%	44%	67%
Residential Neighborhood Level	gated residential neighborhood	100%	56%	67%
	population density	5.65%	4.42%	4.91%
	building floors	50%	67%	60%

**Table 7:** Binary Logistic Regression Analysis of guardians on CIAs permissions.

Variable		Coef.	Std. Err.	Wald	*p* Value	OR	95% CI	
							Upper	Lower
Places where children’s safety incidents occurred	entrance of the residential neighborhood	−0.035	0.282	0.015	0.902	0.966	0.556	1.679
	parking lot	−0.097	0.305	0.102	0.75	0.907	0.499	1.651
	square	−0.058	0.299	0.038	0.845	0.943	0.525	1.693
	waterfront area	−0.55	0.335	2.689	0.100 *	0.577	0.299	1.113
	activity space	−0.486	0.293	2.759	0.097 *	0.615	0.346	1.091
Ways to supervise children	safety education	1.452	0.262	30.587	0.000 ***	4.27	2.553	7.142
	taking mobile phones or positioning wristbands	1.938	0.417	21.641	0.000 ***	6.944	3.069	15.71
	looked after by acquaintances	0.602	0.903	0.445	0.505	1.827	0.311	10.724

Dependent variable: whether allow children to play outside independently or not; OR, odds ratio; 95% CI, 95% confidence interval. *** *p* < 1%; * *p* < 10%.

**Table 8:** Analysis of the correlation between CIAs and the built environment.

Category	Variable	Normality	Children Active Independently	
		*p*	r	*p*
Site Level	site area ^①^	0.343	−0.272	0.327
	activity space area ^①^	0.36	−0.298	0.280
	density of entrances and exits ^②^	0.001 ***	−0.122	0.666
	permeability coefficient of site boundary ^①^	0.346	−0.19	0.498
	rate of tree coverage ^②^	0.015 **	0.582	0.023 **
	rate of shrub coverage ^②^	0.005 **	0.209	0.454
	rate of soft and hard paving ^②^	0.022 **	0.527	0.044 **
	functional mix degree ^①^	0.426	−0.617	0.014 **
Facility Level	density of seats ^②^	0.000 ***	0.354	0.196
	diversity of activity facilities ^②^	0.000 ***	0.551	0.033 **
Management Level	traffic safety ^③^	-	0.327	0.234
	Security ^③^	-	0.063	0.824
	Sanitation ^③^	-	0.593	0.020 **
	facility safety ^③^	-	0.622	0.013 **
Residential Neighborhood Level	gated residential neighborhood ^③^	-	0.687	0.005 ***
	population density ^①^	0.063 *	0.56	0.030 **
	Building floors ^③^	-	−0.313	0.378

^①^ Pearson Correlation; ^②^ Spearman Correlation; ^③^ Independent-Samples *t*-test; *** *p* < 1%; ** *p* < 5%; * *p* < 10%.

## Data Availability

The data are not publicly available due to privacy and security concerns.
